# When stakeholder orientations matter: Modelling employee orientation, shareholder orientation and supply chain orientation as necessary and sufficient conditions for firm performance

**DOI:** 10.1016/j.heliyon.2023.e20359

**Published:** 2023-09-21

**Authors:** Innocent Senyo Kwasi Acquah, Kassimu Issau, Rebecca Dei Mensah, Francis Vanderpuye

**Affiliations:** a Department of Marketing and Supply Chain Management, School of Business, University of Cape Coast, Ghana; b Department of Human Resource Management, School of Business, University of Cape Coast, Ghana; c Institute for Oil and Gas Studies, Faculty of Social Science, University of Cape Coast, Ghana

**Keywords:** Supply chain orientation, Employee orientation, Shareholder orientation, Firm performance, PLS-SEM, Necessary condition analysis

## Abstract

Businesses operate in an increasingly dynamic environment requiring that they strike a delicate, healthy, and equitable balance among the interests of their numerous stakeholders. This study examined how employee orientation and shareholder orientation influence supply chain orientation and how employee orientation, shareholder orientation, and supply chain orientation influence firm performance. The study used a sample of 265 respondents and applied both linear and triangular data analysis techniques in Partial-Least Squares Structural Equation Modelling (PLS-SEM) and Necessary Condition Analysis (NCA) to examine how employee orientation, shareholder orientation, and supply chain orientation influence the performance of oil marketing companies (OMCs) in Ghana. The PLS-SEM results revealed that employee orientation and shareholder orientation are significant predictors of supply chain orientation. The NCA results revealed that employee orientation is the most important for supply chain orientation. Also, from the PLS-SEM results, employee orientation and supply chain orientation predict firm performance, but shareholder orientation does not. However, the NCA results suggest that all three orientations are necessary for firm performance and highlight supply chain orientation as the most important for firm performance. This study is the first to examine how employee orientation and shareholder orientation influence supply chain orientation, besides how the three orientations influence firm performance from both sufficiency and necessity theory perspectives. The study also uncovers supply chain orientation as an underlying process through which employee and shareholder orientation enhances firm performance.

## 1 Introduction

More and more, businesses realise that attending to their diverse stakeholders' needs within and outside the supply chain is a prerequisite for performance. Stakeholder theory (ST) suggests that it is the firm's responsibility to strike an equitable balance amongst the interests of all parties, including shareholders (known as shareholder orientation), employees (known as employee orientation), and other supply chain participants (supply chain orientation) [1]. This is particularly evident in the downstream petroleum sector, where the swift flow of petroleum products through very fluid supply chain operations is critical for customer satisfaction in terms of price and delivery speed references [[2], [3], [4]]. Also, shareholders' attitudes in terms of constant investments in the firms' operations and employee attitudes toward the firm are critical for survival [5,6]. Managing shareholder interest denotes shareholder orientation, while employee orientation signifies an employee-focused organizational climate [[6], [7], [8]]. Yet, achieving this delicate balance among these three orientations has not been easy. Supply chain managers' report mixed perceptions of how employee orientation, shareholder orientation and supply chain orientation contribute to a firm's performance [[9], [10], [11], [12], [13]].

According to Refs. [14,15], supply chain orientation denotes “the recognition by a company of the systemic, strategic implications of the activities and processes involved in managing the various flows in the supply chain”. It emphasizes strategic awareness and embracing supply chain management within an individual supply chain [16,17]. Accordingly, a supply chain-oriented organization is cognizant of the consequences of managing supply chain flows of products, information, and finance [[18], [19], [20]].

However, satisfying the interest of internal and external stakeholders is difficult because they have competing interests in the short term. Hence, a concentration on meeting only one group's needs may be at the expense of the others [21,22]. Employee orientation describes a firm's employee-focused behaviour [12,23], which has been shown that employee-focused behaviour provides resources that the firm uses in connection with other resources from the external environment for increased firm performance [7,8,11,12]. Since employees provide essential services that enable firms to improve their performances, they care about how these organisations reciprocate this gesture while harmonising their values with their employers [23,24].

While prior research investigated the effects of the individual orientations (employee, shareholder, supply chain) on firm performance, none of these studies has done a holistic assessment of the effects of these orientations on firm performance, for example, references [8,10,12,[25], [26], [27]]. However, ST requires that firms strike a balance in attending to the interests of all their stakeholders [2,22,[28], [29], [30]]. Accordingly, focusing only on direct effects does not give a comprehensive picture of how employee orientation, shareholder orientation and supply chain orientation relate to firm performance. Hence, by not investigating the effect of these orientations on firm performance in a single holistic model, we miss insight into how ST can be used to explain the effects of these orientations on firm performance.

Furthermore, prior studies on employee orientation, shareholder orientation and firm performance failed to investigate the underlying processes that explain these relationships [11,31]. As ST suggests, catering for the interest of a set of stakeholders to the detriment of other stakeholders creates a situation where the neglected group uses its power against the firm, resulting in adverse consequences that negate the gains made from such prioritisation (*e.g.,* employees and shareholders).

Nonetheless, resource-based theory suggests that organisations have limited resources compared to the challenges confronting them [32,33]. Therefore, managers are required to prioritise the use of their scarce resources to ensure that priority is given to antecedents that provide the best outcome. Moreover, since firms do not have unlimited resources, besides all orientations not contributing equally to performance improvements, it makes sense to investigate and identify not only the orientations that are necessary for firm performance but also the levels of these orientations that are required for specific desired levels of firm performance. Moreover, prior researchers [[34], [35], [36], [37], [38], [39], [40], [41]] suggest that investigating the relationships between constructs based solely on sufficiency logic fails to paint an accurate picture on the relationships amongst the predictor and outcome constructs. This is because, though some of the exogenous constructs may not be significant predictors of their endogenous counterparts, they may, however, be necessary conditions for producing the outcome. Hence, without a minimum level of this necessary condition, achieving the outcome is not possible [42].

Therefore, developing a more nuanced understanding of how these three orientations produce performance outcomes is a priority for supply chain academics and practitioners [43]. Accordingly, we attempt to bridge this gap with the objectives of (1) assessing the effect of employee orientation and shareholder orientation on supply chain orientation, (2) examining the effect of employee orientation, shareholder orientation, and supply chain orientation on firm performance (3) investigating the mediating roles of supply chain orientation on the effects of (a) employee orientation and (b) shareholder orientation on firm performance and (4) ascertaining the required levels of (a) employee orientation and shareholder orientation to achieve the desired level of supply chain orientation and (b) employee orientation, shareholder orientation and supply chain orientation to achieve the desired level of firm performance. To achieve these objectives, we used PLS-SEM to explore the research questions (1, 2, & 3), which are based on net effects and sufficiency logic, and reference [42] Necessary condition analysis (NCA) to explore research question 4, which is based on necessity logic.

Our study makes three major contributions to the management literature. Firstly, we establish the relationship between the three orientations (employee, shareholder, and supply chain) and firm performance. Our model proposes that employee orientation, shareholder orientation and supply chain orientation directly influence firm performance. Secondly, we provide empirical support for the mediating role of supply chain orientation as a new source of heterogeneity through which employee orientation and shareholder orientation influence firm performance and explain, theoretically, the types of mediation thereof. Thirdly, we make an original contribution to the literature by using two separate but complementary data analytic approaches (i.e., PLS-SEM and NCA) to explore, identify and validate employee orientation, shareholder orientation, and supply chain orientation as both necessary and sufficient conditions for firm performance. The remainder of this paper proceeds with the theoretical background and hypothesis, method, PLS-SEM results, NCA results, discussions, and conclusions.

## 2 Theoretical lens and hypothesis development

### 2.1 Stakeholder theory

Stakeholders are those who have the capability to influence or be influenced by an organisation's activities. As reference [44] puts it, “Stakeholders can affect or be affected by the achievement of the organisation's objectives.” Stakeholders can be a person or a group whose operations affect or are affected by the focal operations. There are two key qualifications criteria for one to qualify as a stakeholder. First, stakeholders should be people or groups who have some legitimate claim on the and second, the interest they hold should be valuable to deserve the organisation's attention. As a result, organisations that excel at managing their relationships with their stakeholders tend to perform better than others who do not. In light of the importance attached to stakeholders within the ST, prior researchers have sought to identify and classify them. The first classification of stakeholders into internal and external stakeholders was by Ref. [44]. He claimed that stakeholders such as employees and suppliers represent internal and external stakeholders, respectively. However, another classification which sorts to group stakeholders into those with the potential to threaten or harm versus those with the potential to cooperate or collaborate was put forth by Ref. [45]. On the other hand, reference [46] grouped stakeholders into normative stakeholders and derivative stakeholders. For him, Normative stakeholders are those whom the organisations have some moral obligations towards (for example, customers and employees), while derivative stakeholders are those whose actions either affect the organisations or the organisation's relationships with the normative stakeholders (your competitors and the public).

Stakeholders are, therefore, groups or individuals who affect or are influenced by the objectives of an organisation [33]. Even though firms seek to maximise value through profit creation, reference [47] suggests that the raison d'etre of businesses goes beyond profit maximisation to include the creation of value for their stakeholders, be it internal, external, normatives or derivatives. Hence, any management activities that are underpinned by the stakeholders' theory will not only provide profit for the firm but will also enhance value creation for the stakeholders [33]. Hence, while shareholder orientation prioritises short-term profitability, shareholder orientation enables firms to adopt a wider and more integrated view that leads to better value creation for all stakeholders. To this end, adopting a stakeholder orientation where the interest of both employees and shareholders, also referred to as internal or “normatives”, as well as those of the other members in the supply chain, such as suppliers, distributors, and logistics service providers also referred to as external or derivatives will help create better value for all such that derivatives who do not have any legitimate claim on the firm may also not take actions that will negatively affect the firm or its relationship with the other stakeholders.

ST provides a basis for the proposed nexus between stakeholder orientations and performance [48]. It underscores the point that an organisation does not operate in a vacuum but with several interested parties (internal and external) whose conflicting interests need to be satisfied for successful performance [49]. Accordingly, a manager must satisfy the conflicting interests of various stakeholders (constituents) whose actions and inaction influence performance [44]. Underpinned by the principle of stimulus-response, shareholders and employees reciprocate positive actions from the organisation by working harder when they see the organisation interested in their affairs, resulting in enhanced performance. In contrast, the organisation's failure to meet employees' and shareholders' expectations activates these internal stakeholders' negative reactions, resulting in poor performance. Stakeholders endeavour to enhance their interest in relation to others; hence, an organisation's failure to maintain an equitable balance among these conflicting interests sparks the need for aggrieved stakeholders to alter the relational process to achieve a new equilibrium and vice versa [50]. They do this by equating their inputs with the organisation's effort to address things of interest to them, resulting in increased or decreased performance [51]. Following the above argument, we expect stakeholders who perceive that their interest is properly catered to reciprocate that gesture to ensure a new but higher equilibrium [52,53].

### 2.2 Effects of employee orientation on supply chain orientation and firm performance

Organisational performance denotes “the actual output or results of an organisation as measured against its intended outputs in terms of goals and objectives” [54]. For a balanced view, we conceptualise organisational performance as having four dimensions: “customer performance, financial performance, internal process performance, and innovation and learning performance” [55,56]. Customer performance measures the “things that are important to our customers, which will, in turn, impact our financial standing [24,55]. Whereas process performance measures what an organisation does internally for improved financial status, learning and growth performance refers to “the skills, culture, and capabilities” [55] that are needed to ensure internal process performance for customer satisfaction and loyalty. Financial performance, which is the ultimate goal of every business, refers to the financial goals that impact the organisation [55].

Employees in organisations with a long-term orientation have a stronger influence on corporate governance [26]. Also, references [8,27], suggest that employee orientation and customer orientations influence employee performance. Again, when reference [11] analyzed 410 mid-senior-level managers in the UK service industry, they found that employee orientation positively influences customer-based performance due to the critical roles employees play in executing other strategic orientations. Moreover, reference [12] opined those thirteen dimensions of employee orientation, namely “training and development, communication skills, convincing skills, interpersonal skills, knowledge management, grievance handling, organisation's culture development, service climate, adaptive behaviour, customer retention skills, customer profiling, trust and commitment, and suitability of employees” enhanced customer performance in various degrees [13]. From the foregoing, we argue that employee orientation would enhance the performance of firms in Ghana's downstream petroleum sector. Employee orientation deepens the trust between the employees and the organization resulting in higher employee input and commitment [56]. Accordingly, we hypothesise that.

H1 Employee orientation is positively related to (a) supply chain orientation and (b) firm performance.

### 2.3 Effects of shareholder orientation on supply chain orientation and firm performance

Shareholder orientation refers to the degree to which a firm's actions are geared to the achievements of the goals and objectives of its owners. Ideally, firms are required to work in the interest of shareholders. However, different stakeholder interests come into play when major decisions are being made [5]. Hence, a strong stakeholder orientation reduces the trade-offs that a limited pursuit of shareholder orientation imposes on firm innovativeness [6]. Moreover, shareholder orientation improves organisational performance because shareholders support the firm's activities when those actions align with their expectations [57,58].

Furthermore, a key effect analysis of four stakeholder orientations revealed that shareholder orientation's major contribution to firm performance was not supported [53]. Accordingly, they opined that shareholder orientation's influence on business performance might be attributed to market conditions surrounding a firm's operations [53]. We, therefore, argue that one of these market conditions is the firm's supply chain. Supply chain orientation signifies the firm's interest in the members of its supply chain, such as customers, suppliers and distributors. Prior research has established a positive relationship between supply chain orientation and firm performance. In that, when the firm prioritises the members of its supply chain, these members replicate this gesture, enhancing a firm's value [58,59]. Therefore, since shareholder orientation influences supply chain orientation and firm performance [5,6], and supply chain orientation also predicts firm performance [15,17,19], we contend that shareholder orientation would predict supply chain orientation and firm performance. Accordingly, we hypothesise that;

H2 Shareholder orientation is positively related to (a) supply chain orientation and (b) firm performance.

### 2.4 Supply chain orientation and firm performance

Supply chain management literature has emphasised the importance of supply chain members, such as suppliers and customers, in enhancing firm performance [5,6,58]. By paying attention to and promoting their interests, both upstream (*e.g.,* suppliers) and downstream (*e.g.,* distributors), supply chain partners can contribute enormously to the successful execution of supply chain strategies [60,61]. The firm's ultimate corporate image and goodwill is also enhanced by the sharing of timely and accurate information, fair negotiations and the recognition of suppliers' need to make profits and ensure that only products of value are channelled through to the customer. Accordingly, a pull rather than a push supply chain strategy promotes this agenda by signifying respect for the customer and giving effect to the cliché that “the customer is king”. Previous literature has also suggested that supply chain orientation predicts the performance of manufacturing firms [15,17,19,59]. Likewise, supply chain orientation has been found to significantly influence halal SMEs' performance [ [62,63]. Based on the above, we hypothesise that;

H3 Supply chain orientation is positively related to firm performance.

### 2.5 The mediating role of supply chain orientation

Business organisations do not only deal with internal stakeholders but also external stakeholders. These external stakeholders (*e.g.,* suppliers, logistics service providers and customers) are within the organisation's supply chain. The importance of the stakeholders within the supply chain cannot be overemphasised [60]. Generally, supply chain members are regarded as external stakeholders with at least a contractual relationship with the organisation. A supply chain orientation refers to “the extent to which chain members have a predisposition toward viewing the supply chain as an integrated entity and on satisfying the supply chain needs in an integrated way” [64]. We focused on supply chain orientation as a composite construct. We contend that the whole supply chain serves as a boundary condition that influences the relationship between an employee, shareholder orientation, and firm performance.

This predisposition can arise when supply chain members develop shared values and beliefs centred on the overall supply chain's importance, not just on their specific functions. We draw on ST as the basis for the proposed mediating role of supply chain orientation. Central to the ST is the assumption that organisations need to balance the competing interests of their stakeholders (internal and external). Members of the supply chain are external stakeholders whose interests need to be balanced against the internal stakeholders [65]. Employees do interphase with external stakeholders such as customers and suppliers, and how they feel influences their behaviour and attitudes towards them. Accordingly, when employees perceive the firm and their managers to have their interests at heart, they become happy and work even harder [53]. From the preceding arguments and the ST, we expect that promoting the interest of employees will result in happy employees who perform to the satisfaction of external stakeholders within the firm's supply chain.

So far, we argue that employee orientation and shareholder orientation are positively related to supply chain orientation and that supply chain orientation is directly related to firm performance [8,10,15,17,19,27,59,60,62,63]. Also, based on the theoretical underpinnings of ST [2,22,[28], [29], [30]], we argue that the relationships between employee orientation and shareholder orientation on one hand and firm performance on the other hand should be explained via their effects on supply chain orientation. Further, prior research based on the ST suggests that supply chain orientation is an important mechanism through which employees and shareholders enhance firm performance [2,22,29,30]. The foregoing discussion leaves us to suggest that a relevant mediating process by which employee orientation and shareholder orientation affect firm performance is via enhancement in supply chain orientation elements such as supplier orientation and customer orientation. Thus, we hypothesised that the basis for the influence of employee orientation and shareholder orientation on firm performance is because they both influence supply chain orientation – a critical cause of a firm's performance outcomes. This thought is accordingly articulated as follows;

H4 Supply chain orientation mediates the relationship between (a) employee orientation and firm performance and (b) shareholder orientation and firm performance.

### 2.6 Orientations as necessary conditions

**Fig. 1:**
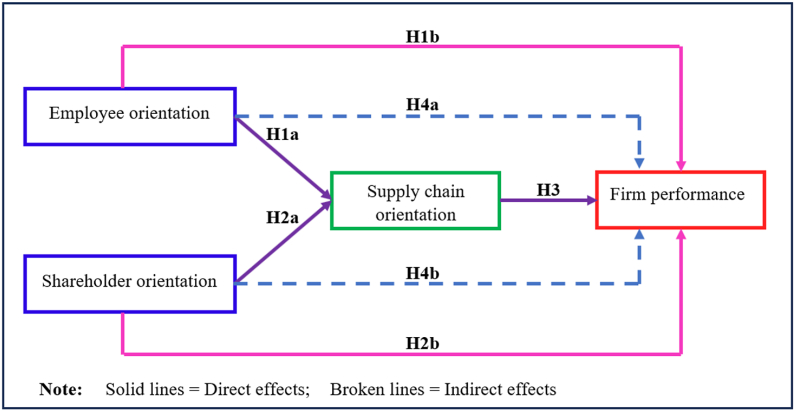
Conceptual framework.

The literature on the nexus between employee, shareholder and supply chain orientations on firm performance proffers a symmetric relationship between these orientations and firm performance. Specifically, prior researchers [5,6,53,58] used sufficiency logic to opine that employee orientation reference [8,26,27], shareholder orientation [53], and supply chain orientation [5,6,58] are sufficient for firm performance. However, identifying the orientation(s) that is necessary for firm performance is equally imperative. Also worthy is ascertaining the necessary levels of these orientations, thus serving as bottlenecks for achieving firm performance. Therefore, if the firm fails to provide the necessary level of orientation, the firm will not perform. This suggests that if a firm fails to ensure that a certain level of employee orientation, shareholder orientation or supply chain orientation, for instance, a high level of performance cannot be achieved. Accordingly, this study proposes that, first, employee orientation (a) and shareholder orientation (b), are necessary but not sufficient drivers of firm performance supply chain orientation and second, employee orientation (a), shareholder orientation (b), and supply chain orientation (c) are necessary, but not sufficient drivers of firm performance. Fig. 1 presents the conceptual model of the study.

## 3 Method

### 3.1 Sample and procedure

We used a cross-sectional survey sample of managers from the OMCs in Ghana between March to May 2022. The surveys were distributed to the participants who know and work in the respective firms' operations, logistics and supply chain functions. Out of 500 questionnaires distributed, 282 were returned, resulting in a response rate of 56.4%. However, 17 responses were incomplete; hence, they were deleted, leaving a useable sample of 265 responses. This response rate was achieved because we sent several reminders through phone calls and emails to the respondents. Appendix A provides information on the respondents’ demographic characteristics.

The association of OMCs granted consent for the data collection upon submitting an introductory letter from the School of Business, University of Cape Coast. Further, a cover letter describes each questionnaire package's purpose and confidential nature. Each respondent was also required to sign an informed consent letter before participating in the study. The questionnaire was self-administered through the drop-and-pick method by trained research assistants who volunteered and distributed the questionnaire packages to the key informants in each organisation.

We used the approaches proposed by Refs. [66,67], and [68] to ascertain the required sample size for the study. Using the [66] power table, the minimum sample size for our model with 3 exogenous constructs, an alpha level of 5%, and a minimum expected R^2^ of 0.1 is 124. Going by reference [67] approach, the reasonable minimum sample size prior to data collection is 146 and 160 based on the gamma-exponential and inverse square root methods, respectively. Hence, since the inverse square root method produces a higher sample size than the gamma-exponential method, this study's reasonable minimum sample size will be 160. Going by the approach proposed by Ref. [68], we conducted a priori and post-hoc power analyses to determine and confirm the minimum sample size for the study. The prior power analysis was to ascertain the appropriate sample size before data collection, with an effect size, significance level and power values of 0.15, 0.05, and 0.90, respectively, along with three exogenous constructs. This prior power analysis yielded a sample size of 73. Since a useable sample size of 265 was obtained after data cleaning, we performed a post-hoc power analysis [69] to ascertain whether the useable sample size of 265 corresponds to the power level used for a priori power analysis. Results of the post-hoc analysis revealed a power of 0.999. Hence, our useable sample size of 265 was deemed appropriate for the study because it was higher than the largest minimum sample sizes that emerged from the three approaches proposed by Refs. [[66], [67], [68]].

### 3.2 Measures

Employee orientation was measured with four items adapted from Ref. [52]. Sample items include “we have regular staff meetings with employees”, “we have regular staff appraisal in which we discuss employees' needs”, “we survey staff at least once a year to assess their work attitudes”, and “we try to find out the true feelings of staff about their jobs”. Shareholder orientation was measured with five items adapted from Ref. [52]. Sample items include “We regularly carry out public relations aimed at stakeholders”, “our objectives are driven by creating shareholder wealth”, “designated managers are responsible for satisfying shareholders’ interests”, and “we regularly compare our share value with that of our competitors” and “We have regular meetings with shareholder”.

The supply chain orientation (SCO) scale, which was adapted from Ref. [52] consists of 60 items, measuring six dimensions: Customer orientation (*e.g.,* “We constantly monitor our commitment to serving customer needs as part of our value chain activities”), Competitor orientation (*e.g.,* “We constantly monitor our commitment to understanding competitors as a part of our value chain activities”). Supplier orientation “we constantly monitor our commitment to understanding suppliers as a part of our value chain activities”), logistics orientation, “we constantly monitor our commitment to understanding our logistics activities as a part of our value chain activities”), operations orientation (“we constantly monitor our commitment to understanding our operations management activities as a part of our value chain activities”), and value-chain coordination (“we constantly monitor our coordination of value chain functions”).

Firm performance was measured with 16 items adapted from Ref. [52]. It measures four dimensions of firm performance as follows: (a) Customer performance (*e.g.,* “We achieved a high degree of customer satisfaction in the last year” and “we kept a large number of existing customer market share in the last year).” (b) Financial performance (*e.g.,* “we achieved revenues above our stated objective in the last year” and “we achieved sales above our stated objective in the last year”. (c) Internal process performance (*e.g.,* “the speediness of our supply chain processes improved in the last year” and “the quality of our supply chain processes improved in the last year”), and (d) Innovation and learning performance (*e.g.,* we significantly enhanced our marketing skills compared with last year” and “we significantly enhanced our logistics skills compared with last year”). We measured all items on a five-point Likert scale, ranging from (1) strongly disagree to (7) strongly agree, and the descriptive statistics for all construct is presented in Appendix B.

### 3.3 Bias testing

Since we used a survey and adopted a measurement approach that is susceptible to nonresponse bias and common method bias (CMB), we took appropriate steps to check for both. For nonresponse bias, we employed an independent samples *t*-test by comparing early responses to late responses and found that at a significant level of 0.05, no differences between the two groups of responses. Accordingly, we conclude that nonresponse bias is not a problem for the hypothesised model. For common method bias, we tried to minimise it by adopting both procedural and statistical approaches. For the procedural approach, we ensured that our language and questionnaire were simple and short to ensure that our questions were not invasive and sensitive but guaranteed anonymity and voluntary participation. For the statistical approach, we assessed the possibility of CMB using Harman's one-factor test [70]. The resultant principal component analysis on all items used in the study (Appendix C), with principal axis factoring as the extraction method, shows that a single factor accounted for 34.486% of the variance, and since this is far less than 50%, we conclude that CMB is not a problem in this study.

### 3.4 Analysis strategy

Since the study is premised on both sufficiency and necessity logic, the corresponding analysis approaches (*i.e.,* SEM and NCA) were employed in two sequential phases to determine whether these orientations are sufficient or necessary for firm performance. Whereas the first phase was for the sufficiency analysis, the second phase focused on the necessity analysis. We considered the variance-based approach to structural equation modelling for the sufficiency analysis as the most appropriate for the study. This is because other statistical methods are accustomed to analysing relationships between constructs individually [71]. Furthermore, this research is more attuned to theory exploration rather than theory confirmation to predict firm performance or identify key driver constructs (employee orientation, shareholder orientation and supply chain orientation) of firm performance [71]. Finally, variance-based PLS-SEM was chosen because the study sought to maximise the explained variance in the endogenous construct. The analysis was in two main stages. In the first stage, we specified both the measurement and the structural model, examined the reliability and validity of the constructs, and finally examined the structural path coefficients, VIF, In-sample prediction statistics (R^2^, R^2^ adjusted & Q^2^) and out-of-sample prediction statistics (Q^2^predict, RMSE, MAE & MAPE). The mediation analysis followed reference [72] approach to mediation analysis. In the second phase, we employed reference [42] necessary condition analysis (NCA) to determine whether employee orientation, shareholder orientation and supply chain orientation are necessary for firm performance. Necessary condition analysis is an analytical procedure underpinned by the necessity logic and provides a more nuanced understanding of necessary condition analysis, identifying not only necessary conditions but also the degree to which each condition is necessary. It helps identify conditions that constitute bottlenecks to achieving an outcome, in that, a necessary condition does not guarantee the outcome but does enough to constrain it, thereby serving as a bottleneck to achieving the outcome. Accordingly, NCA is an appropriate complementary tool for regression-based analytical tools.

### 3.5 Robustness check

Based on the recommendations of reference [73] on the robustness check in PLS-SEM, we assessed endogeneity, heterogeneity, and nonlinear effect. Endogeneity, which threatens the validity of PLS-SEM results, may arise from Omitted variables bias, Simultaneity, Measurement error, or Common Method Variance [[73], [74], [75]]. We adopted reference [76] procedure by applying the Gaussian copula approach [77] to assess the possibility of endogeneity in our data. The results show that none of the exogenous constructs, namely employee orientation, shareholder orientation and supply chain orientation, had a significant Gaussian copula (i.e., P value > 5%). More specifically, the analysis yielded non-significant copulas of −0.509 (P value = 0.073), −0.08 (P value = 0.786), and 0.256 (P value = 0.364) for Employee orientation, supply chain orientation and shareholder orientation, respectively. Additionally, the combination of Gaussian copulas contained in the model was assessed and produced non-significant results (Appendix D). Accordingly, we concluded that endogeneity is absent from this study, thereby supporting the robustness of the structural model results for the study.

In checking for unobserved heterogeneity, we performed the Finite Mixture PLS procedure (FIMIX-PLS). Prior to performing the FIMIX-PLS, one has to ascertain the number of segments to extract. Hence, we used reference [66] table to determine the minimum sample size required to produce valid results for a model with a maximum of three exogenous constructs pointing at one endogenous construct where the alpha level is 5%, and the minimum expected coefficient of determination of 20% is 59. This translates into 4.49 segments when we divide the sample of 265 by 59, thereby suggesting a four-segment solution. However, based on the inverse square root method [67], a minimum sample size of 160 is required for each segment. This translates into 1.66 segments when we divided the sample of 265 by 160, thereby suggesting a one-segment solution. Nonetheless, to be sure that unobserved heterogeneity is not an issue in this study, we proceeded to perform FIMIX-PLS with the four-segment solution. The results of the fit indices for the four-segment solution are presented in Appendix E1. In determining the number of segments to retain, the approach proposed by Ref. [71] was applied where, except for EN, the optimum solution denotes the number of segments having the lowest values for FIMIX-PLS in terms of AIC3, AIC4, BIC, CAIC, HQ, and MDL5. According to the criteria, the optimum number of segments should be higher than the number of segments suggested by MDL5, which, based on our results, should be more than two segments further, since the lowest of AIC3, AIC4, BIC, CAIC, HQ are owned by segment two. Though most of the fit indices pointed to a two-segment solution, they failed to agree on a specific number of segments. Hence, we proceeded to the next criteria by evaluating the sample sizes for each segment [67,71]. suggested that the sample sizes for the segments to be used should meet the minimum sample size requirement for running PLS-SEM. We also applied the minimum sample size criteria in determining the number of segments to use. From the sample analysis done earlier, a four-segment solution is not feasible because the sample size for the fourth segment falls below 59. Likewise, because the relative sample size for the third segment in a three-segment solution is 37, a three-segment solution is also not feasible, leaving us with a two-segment solution.

When AIC3 and CAIC point to the same number of segments, the results will likely converge to produce the specific number of segments [78]. However, from our results, AIC3 suggests a four-segment solution while CAIC suggests a two-segment solution, signalinging that the results are not likely to produce the specific number of segments to be used for FIMIX-PLS. This confirms why the remaining fit indices failed to point at a specific number of segments. First, AIC4 and BIC, which are good at determining the number of segments in FIMIX-PLS and are clustered around the entropy criterion, suggested a two-segment solution. Second, per the minimum sample size requirement, a two-segment solution is the most appropriate as the sample sizes for the third segment in a three-segment solution, along with the third and fourth segments in a four-segment solution, fell below the minimum sample size requirement. Third, the lowest figure for MDL5 is owned by segment two however, because the MDL5 criterion tends to underestimate the number of segments, it is interpreted to imply more segments than the segment with the lowest MDL5. Hence, even though the lowest figure for MDL5 resides in segment two, it implies three or more segment solutions [73]. Reading all the criteria together, the metrics produced contradictory results (Appendix E2) and failed to unequivocally indicate the specific number of segments to be extracted, thereby, suggesting a one-segment solution. This is because (a) AIC3 and CAIC suggest different segment solutions (i.e., a four-segment and two-segment solution, respectively (b) MDL5 suggests more than a two-segment solution, which can be a three or a four-segment solution (c) AIC4 and BIC suggest a two-segment solution. Accordingly, and in line with reference [73], we assume that unobserved heterogeneity does not substantially influence the results of a one-segment solution (i.e., a full data set) and conclude that the results meet the robustness criteria.

We employed the two-stage approach recommended by Ref. [78] to examine the non-linear effects between the constructs. The results from the test of quadratic (non-linear) effects [73] show that none of the non-linear (quadratic) effects was significant (see Appendix F), accordingly confirming the validity and robustness of PLS-SEM results.

## 4 PLS-SEM results

### 4.1 Measurement model results

Since all constructs in the model were measured reflectively, the resultant measurement model was assessed for internal consistency reliability and construct validity (convergent and discriminant) using SmartPLS v3.5. We assessed internal consistency reliability using Cronbach's alpha (CA) and Composite Reliability (CR). Convergent validity was assessed using item loadings and Average Variance Extracted (AVE). The measurement model results (Table 1) show that CA values of all constructs exceeded the minimum threshold of 0.7 reference [78] while all CR values exceeded the minimum threshold of 0.7. Accordingly, the CA and CR values for all constructs confirm the internal consistency reliability of the constructs [79]. Also, item loadings were above the recommended threshold of 0.7, confirming the indicator reliability of the items used to measure the constructs in the model. Additionally, the results (Table 1) further show that the AVEs for all constructs used in the model were above the recommended minimum of 0.5, confirming the convergent reliability status of the constructs [79]. Therefore, the model meets the internal consistency reliability and convergent validity requirement as espoused by reference [79].

**Table 1:** Measurement model results.

**Criteria**	***Employee orientation***	**Shareholder orientation**	**Supply chain orientation**	**Firm performance**
Factor Loadings (Min - Max)	0.826–0.895	0.832–0.859	0.878–0.920	0.873–0.909
Cronbach's alpha	0.873	0.898	0.951	0.917
Rho_A	0.878	0.899	0.952	0.918
Composite reliability	0.913	0.925	0.961	0.942
Average Variance Extracted	0.725	0.711	0.805	0.801
**Fornell-Larcker Criterion**				
**Construct**	**1**	**2**	**3**	**4**
1 Employee Orientation	**0.851**			
2 Shareholder Orientation	0.702	**0.843**		
3 Supply Chain Orientation	0.680	0.660	**0.879**	
4 Firm Performance	0.657	0.632	0.827	**0.895**
Note: Values in bold denote the square root of AVEs; Off-diagonal values denote the correlation between**Constructs.**				
**Heterotrait-Monotrait (HTMT) Ratio** [80]				
**Construct**	**1**	**2**	**3**	**4**
1 Employee Orientation				
2 Shareholder Orientation	**0.789** [0.6880.868]			
3 Supply Chain Orientation	**0.744** [0.651; 0.815]	**0.712** [0.587; 0.802]		
4 Firm Performance	**0.729** [0.638; 0.802]	**0.695** [0.588; 0.780]	**0.884** [0.823; 0.929]	

Note: *Items in bold = HTMT Values; Items in parenthesis = lower and upper bound of 95% biased corrected and accelerated confidence intervals*.

Also, discriminant validity was assessed using the Fornnel-Lacker criterion and the HTMT ratio. Per the results (Table 1), the square root of the AVE's of all constructs was higher than the construct correlations. Likewise, most of the HTMT values were below the cut-off point of 0.90, with only two of them going beyond the threshold. However, since none of the biased correlated confidence intervals (for these HTMT values) contained 1, the model is deemed to have met the HTMT criterion for determining discriminant validity. Accordingly, using the Fornel-Lacker criterion and the HTMT, we conclude that the model achieved discriminant validity. Hence, the next section assesses the structural model.

### 4.2 Structural model results

The structural model results (Table 2) assessed collinearity (VIF), in-sample prediction (via R^2^, f^2^ and Q^2^), out-of-sample prediction (via PLSpredict), and structural model path coefficients. The subsequent path analysis is divided into (1) analysis of direct effects and (2) analysis of indirect effects (mediating analysis).

**Table 2:** Structural model results.

Direct effects	Coefficient	Std.	T value	P value	BCaCI	f^2^	VIF	Decision
*H1a:* EMO ➔ SCO	0.427	0.078	5.444	0.000	0.279; 0.582	0.196	1.973	Supported
*H1b:* EMO ➔ FMP	0.133	0.059	2.280	0.023	0.021; 0.249	0.025	2.359	Supported
*H2a:* SHO ➔ SCO	0.361	0.090	4.007	0.000	0.178; 0.524	0.140	1.973	Supported
*H2b:* SHO ➔ FMP	0.092	0.058	1.596	0.111	−0.025; 0.202	0.013	2.249	Not Supported
H3*:* SCO ➔ FMP	0.675	0.052	12.876	0.000	0.567; 0.773	0.726	2.120	Supported
**Indirect effects**	**Coefficient**	**Std.**	**T value**	**P value**	**BCaCI**	**Decision**	**Type of Mediation**	
EMO ➔ SCO ➔ FMP	0.288	0.056	5.133	0.000	0.188; 0.405	Supported	Partial Complementary	
SHO ➔ SCO ➔ FMP	0.243	0.066	3.686	0.000	0.120; 0.378	Supported	Full	
**In-sample prediction results**						***R***^***2***^	***R***^***2***^***Adjusted***	***Q***^***2***^
Construct						0.528	0.525	0.623
Firm Performance						0.704	0.701	0.744
**Out-of-sample prediction Statistics**	**RMSE**		**MAE**		**MAPE**		**Q**^**2**^**_predict**	
	**PLS-M**	**LR-M**	**PLS-M**	**LR-M**	**PLS-M**	**LR-M**	**PLS-M**	**LR-M**
*sco1*	0.786	0.795	0.625	0.627	24.906	24.454	0.462	0.450
*sco2*	0.788	0.795	0.617	0.606	23.500	22.850	0.427	0.418
*sco3*	0.861	0.870	0.663	0.671	25.768	26.293	0.347	0.333
*sco4*	0.857	0.884	0.668	0.697	26.268	27.749	0.374	0.333
*sco5*	0.821	0.847	0.659	0.674	26.929	27.202	0.452	0.417
*sco6*	0.847	0.875	0.641	0.660	25.484	26.417	0.390	0.350
*fmp1*	0.895	0.897	0.668	0.675	26.376	26.606	0.346	0.344
*fmp2*	0.819	0.848	0.613	0.624	24.061	24.418	0.400	0.356
*fmp3*	0.813	0.846	0.612	0.636	23.054	23.727	0.394	0.344
*fmp4*	0.856	0.875	0.669	0.685	25.980	26.084	0.377	0.350

Note: EMO = Employee orientation; SHO = Shareholder orientation; SCO = Supply chain orientation; FMP = Firm performance; RMSE = Root Mean Squared Error; MAE = Mean Absolute Error; MAPE = PLS-M = Partial Least Squares Model; LR-M = Linear Regression Model; BCaCI = Biased Corrected and Accelerated Confidence Interval.

#### 4.2.1 Predictive statistics (in-sample and out-of-sample)

The model explains 52.8% and 70.4% of the variation in supply chain orientation (R^2^ = 0.528) and firm performance (R^2^ = 0.704), respectively. Regarding the effects size of the direct effects represented by the f^2^ values (Table 2), whereas employee orientation and shareholder orientation had medium effects on supply chain orientation, the effects of the three orientations on firm performance were small, with employee orientation having the highest effect (0.162) on supply chain orientation while supply chain orientation had the highest effect (0.7268) on firm performance.

The model was further examined for the predictive relevance of the endogenous constructs in the model. The results (Table 2) further suggest Q^2^ values of 0.623 and 0744 for supply chain orientation and firm performance, respectively. Since all the Q^2^ values are positive and higher than zero, the model's predictive relevance is confirmed. These results (R^2^, f^2^ and Q^2^) suggest that the model has adequate in-sample prediction power (Hair et al., 2020). Lastly, we performed PLSpredict analysis to establish the out-of-sample predictive power of the model. The results (Table 2) indicate that all the Q^2^predict statistics for the PLS model outperformed that of the linear model, and all the indicators of firm performance had lower prediction errors for the PLS-SEM model in comparison with the linear benchmark. Accordingly, the model is deemed to have high predictive power [79,81].

#### 4.2.2 Hypotheses testing

The results for the direct effects are presented in Table 2. According to the results, employee orientation (β = 0.427, t = 5.444, & p = 0.000), and shareholder orientation (β = 0.361, t = 4.007, and p = 0.000) significantly predict supply chain orientation, supporting Hypotheses H1a and H2a respectively. Also, though employee orientation (β = 0.133, t = 2.280, and p = 0.023), and supply chain orientation (β = 0.675, t = 12.876, and p = 0.000) significantly predict firm performance and supporting hypotheses H1b, H2b and H3 respectively, the effect of shareholder orientation (β = 0.092, t = 1.596, and p = 0.111) on firm performance was not significant.

The results (Table 2) further suggest that the indirect relationship between employee orientation and firm performance through supply chain orientation was positive and significant (β = 0.288, t = 5.1333, and p = 0.000), while that between shareholder orientation and firm performance through supply chain orientation was also significant (β = 0.243, t = 3.686, and p = 0.000). Accordingly, H4a and H4b were respectively supported.

## 5 NCA results

**Fig. 2:**
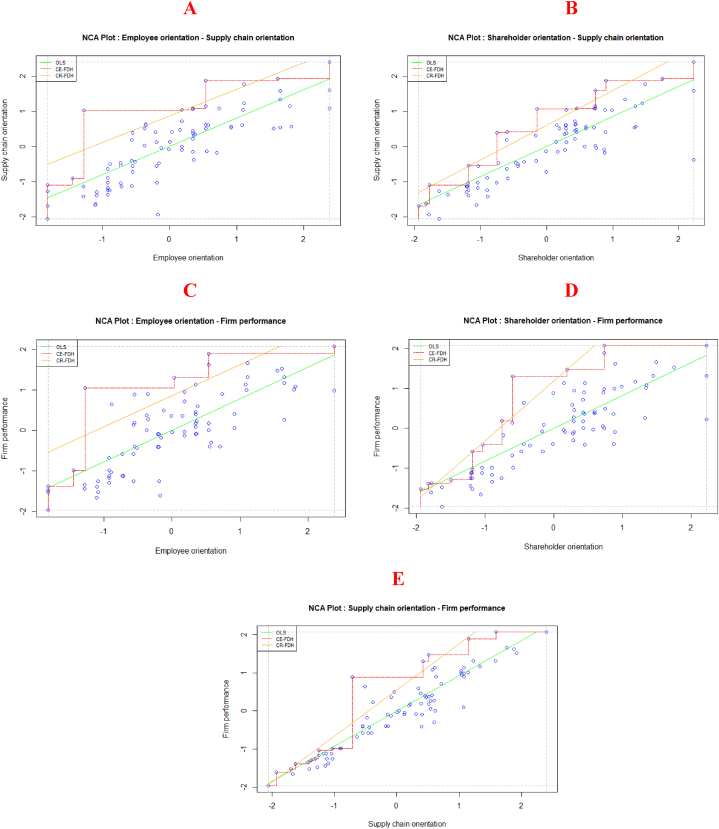
NCA plots.

The NCA analysis was done using the latent variable scores [[82], [83], [84]] from the PLS-SEM analysis as inputs into the NCA package in R v3.03. Though several parameters are produced for both the CE-FDH and CR-FDH ceiling lines, this result evaluates the scatter plots’ effect sizes and their significance, as well as the bottlenecks for each condition and associated outcome. The scatter plots for each condition and associated outcome are presented in Fig. 2. Panel A depicts the relationship between employee orientation and supply chain collaboration, Panel B represents the nexus between shareholder orientation and supply chain orientation. Similarly, Panel C denotes the link between employee orientation and firm performance, while Panel D stands for the connection between shareholder orientation and firm performance. Finally, the link between supply chain orientation and firm performance is presented in Panel E. An observation of which shows an empty space at the upper left corner of each scatter plot in panels A, B, C, D and E, suggesting the likely presence of a necessary condition [82]. Since this empty space is found on each scatter plot for each condition and associated outcome, these conditions are likely necessary conditions for their respective outcomes in the study.

### 5.1 Effect size and significance testing

As recommended by Ref. [42], a random sample size of 10000 was used to examine the effect sizes (d) of the conditions - employee orientation, shareholder orientation and supply chain orientation. As references [42,82] suggest, a condition is necessary when there is (a) theoretical support for it, (b) the effect size is greater than zero and is significant at 0.05. We followed the criteria proposed by Dul 2016 and assessed effect sizes using 0 < d < 0.1 = “small effect”, 0.1 ≤ d < 0.3 = “medium effect”, 0.3 ≤ d < 0.5 = “large effect”, and d ≥ 0.5 = “very large effect”. The results (Table 3) display the effect sizes for employer orientation (d = 0.159, p = 0.000) and shareholder orientations (d = 0.145, p = 0.000) as necessary conditions for supply chain collaboration which are all greater than zero with p values less than 0.05. Therefore, employee orientation and shareholder orientation are deemed necessary for supply chain collaboration because the effect size and significance values have all met the criteria [42,85]. Table 3 further displays effect sizes for employer orientation (d = 0.161, p = 0.000), shareholder orientations (d = 0.141, p = 0.000) and supply chain collaboration (d = 0.145, p = 0.000) as necessary conditions for firm performance, which are all greater than zero with p values less than 0.05. Thus, employee orientation, shareholder orientation and supply chain orientation are accordingly deemed to be necessary for firm performance.

**Table 3:** Effect size and significant testing.

Construct	Method	Accuracy	Ceiling Zone	Scope	Effect size (d)	P value	Condition Inefficiency	Outcome Inefficiency
Employee orientation	CE - FDH	100%	4.703	27.435	0.171	0.000	61.100	6.151
	CR - FDH	98.1%	4.404	27.435	0.161	0.000	63.197	12.760
Shareholder orientation	CE - FDH	100%	4.834	27.046	0.179	0.000	71.522	0.000
	CR - FDH	98.9%	3.817	27.046	0.141	0.000	70.836	3.224
Supply chain orientation	CE - FDH	100%	3.610	17.408	0.207	0.000	75.000	6.565
	CR - FDH	97.0%	2.521	17.408	0.145	0.000	71.297	0.000
**Notes:** “Outcome construct = firm performance; *CE*-*FDH* = *Celling envelopment with free disposal hull*; *CR-FDH* = *Celling regression with free disposal hull*; ^*a*^ 0.1 ≤ d < 0.3 = medium effect”								
Employee orientation	CE - FDH	100%	3.665	27.293	0.134	0.000	51.894	12.357
	CR - FDH	98.9%	4.332	27.293	0.159	0.000	57.339	25.585
Shareholder orientation	CE - FDH	100%	4.658	26.905	0.173	0.000	75.136	4.101
	CR - FDH	98.9%	3.900	26.905	0.145	0.000	70.464	1.845

**Notes:** “Outcome construct = supply chain collaboration; *CE*-*FDH* = *Celling envelopment with free disposal hull*; *CR-FDH* = *Celling regression with free disposal hull*; ^*a*^ 0.1 ≤ d < 0.3 = medium effect”.

### 5.2 Bottleneck analysis

Bottleneck analysis was also performed to determine the conditions constraining the outcomes in the model – supply chain collaboration and firm performance. The ranges of 0.00–0.30, 0.31–0.70, and 0.71–1.0 were used to respectively ascertain the critical values for “low, medium, and high levels” of supply chain orientation and firm performance [83]. Regarding supply chain collaboration as an outcome construct, Table 4 (see the lower part) provides the minimum of employee orientation and shareholder orientation required for a desired level of supply chain orientation. Hence, according to the results (Table 4), achieving a medium to a high level of supply chain orientation, a minimum level of 25.6% for employee orientation and 1.8% for shareholder orientation are necessary. However, for very high levels of supply chain orientation (*e.g.*, 100%), 42.7 and 29.55 of employee orientation and shareholder orientation are necessary. This implies that very high levels of supply chain collaboration can only be guaranteed when the levels of employee orientation and shareholder orientation are at least 42.7% and 29.555, respectively. Similarly, when firm performance is the outcome construct, Table 4 again provides the minimum levels of employee orientation, shareholder orientation and supply chain orientation that are required for desired levels of firm performance. For medium to high levels of firm performance, a minimum level of 12.8% of employee orientation, 3.2% of shareholder orientation and 0.0% of supply chain orientation are necessary. Nevertheless, for very high levels of firm performance (*e.g.*, 100%), 36.8 of employee orientation, 29.2% of shareholder orientation and 28.7 of supply chain orientation are necessary. Therefore, very high levels of firm performance won't occur if certain minimum levels of employee orientation (12.8%), shareholder orientation (3.2%) and supply chain orientation (0.0%) are not achieved.

**Table 4:** Bottleneck levels in (in %) using both CE-FDH and CR-FDH, (NN = Not necessary).

Y Firm Performance (%)	X_1_ Employee Orientation		X_2_ Shareholder Orientation		X_3_ Supply Chain Orientation	
	CE-FDH NC from 6.2%	CR-FDH NC from 12.8%	CE-FDH NC from 0.0%	CR-FDH NC from 3.2%	CE-FDH NC from 6.7%	CR-FDH NC from 0.0%
0	NN	NN	NN	NN	NN	0.3
10	08.1	NN	03.0	02.0	04.3	03.1
20	14.0	03.1	06.8	05.1	24.5	05.9
30	14.0	07.3	06.8	08.1	24.5	08.8
40	14.0	11.5	21.2	11.1	24.5	11.6
50	14.0	15.7	21.2	14.1	24.5	14.5
60	14.0	19.9	21.2	17.1	24.5	17.3
70	14.0	24.1	21.2	20.1	24.5	20.2
80	14.0	28.4	24.9	23.1	25.0	23.0
90	38.9	32.6	28.5	26.2	25.0	25.9
100	38.9	36.8	28.5	29.2	25.0	28.7
**Note**: CE-FDH = Celling envelopment with free disposal hull; CR-FDH = Celling regression with free disposal hull; NN = Not necessary; NC = Necessary condition						
	Y Supply Chain Collaboration (%)	**X**_**1**_ Employee Orientation		**X**_**2**_ Shareholder Orientation		
		CE-FDH NC from 12.4%	CR-FDH NC from 25.6%	CE-FDH NC from 4.1%	CR-FDH NC from 1.8%	
	0	NN	NN	NN	NN	
	10	NN	NN	03.3	02.5	
	20	14.0	NN	06.8	05.5	
	30	14.0	02.5	06.8	08.5	
	40	14.0	08.3	21.2	11.5	
	50	14.0	14.0	21.2	14.5	
	60	14.0	19.7	24.8	17.5	
	70	14.0	25.5	24.8	20.5	
	80	14.0	31.2	24.9	23.5	
	90	14.0	36.9	24.9	26.5	
	100	24.9	42.7	48.1	29.5	

**Note**: CE-FDH = Celling envelopment with free disposal hull; CR-FDH = Celling regression with free disposal hull; NN = Not necessary; NC = Necessary condition.

## 6 Discussion

Grounded in stakeholder theory, the study sought to achieve four main objectives by formulating and testing a model to assess how employee orientation, shareholder orientation and supply chain orientation influence firm performance within the petroleum sector. First, we examined the effect of employee orientation, shareholder orientation and supply chain orientation on firm performance. Secondly, the mediation effect of supply chain orientation on the relationship between employee orientation and firm performance was also assessed. Thirdly, the study investigated the mediation role of supply chain orientation on the relationship between shareholder orientation and firm performance. Fourthly, assessed the necessity of employee orientation, shareholder orientation and supply chain orientation for firm performance. We tested the hypothesised model in two ways by first performing PLS-SEM to assess the direct and indirect effects of employee orientation, shareholder orientation and supply chain orientation on firm performance and second, we performed NCA to explore the degree to which employee orientation, shareholder orientation and supply chain orientation are necessary for firm performance. Whereas the results of the PLS-SEM underscored the statistically significant effects of employee orientation, shareholder orientation and supply chain orientation on firm performance, the NCA findings suggested that all three orientations under study are necessary (albeit to different degrees) for firm performance.

For the direct relationships, the relationship between employee orientation and firm performance was found to be significant, which supports the findings of reference [8,12], and [13]. However, the relationship between shareholder orientation and firm performance was also not significant, which is inconsistent with the findings of reference [6] as well as reference [53]. Further, supply chain orientation was found to be a significant predictor of firm performance, which is consistent with the findings of reference [60 [17], and [59]. The study also found a significant relationship between employee orientation and supply chain orientation, which supports the findings of reference [11,27]. Shareholder orientation also showed a significant effect on supply chain orientation, which aligns with the findings of reference [5,6,58].

For the indirect effects, supply chain orientation was found to mediate the relationship between employee orientation and firm performance, supporting previous studies that suggest that supply chain orientation mediates the effect of employee orientation and firm performance [8,12]. Finally, the results suggest that the relationship between shareholder orientation and firm performance is significantly mediated by supply chain orientation, which indirectly corroborates prior studies that highlight the positive relationship between shareholder orientation and firm performance [6,53].

Regarding the NCA results (Table 4), employee orientation d = 0.159) had the largest effect size on supply chain orientation than shareholder orientation (d = 0.145). Moreover, employee orientation (d = 0.161) had the largest effect size on firm performance, followed by supply chain orientation (d = 0.145) and shareholder orientation (d = 0.141). Interestingly, even though shareholder orientation was not a significant predictor of firm performance in the PLS-SEM analysis, the NCA results (Table 4) suggest that it is a necessary condition for firm performance such that if at least 3.2% of shareholder orientation is not present, firm performance is not guaranteed. Hence, for managers to achieve the desired outcomes, required levels of these conditions should be provided. Moreso, managers will be wasting resources if these conditions are provided beyond the minimum require for the highest level of the outcome because the excess investment does not yield any additional gain.

### 6.1 Theoretical implications

This study examined employee, shareholder, and supply chain orientations in one integrated model. In the stakeholder orientation domain, these three stakeholders are usually not studied together hence prior researchers classify some of the supply chain orientation dimensions under market orientation and studied its link to firm performance whiles the roles of employee orientation and shareholder orientation in enhancing firm performance is largely ignored. Accordingly, this study identified employee orientation, shareholder orientation and supply chain orientation as predictors of firm performance. Therefore, this study might be the first to identify and combine these orientations to explain firm performance, thereby enhancing our understanding of how a firms orientation affects its performance.

First, besides the relationship between shareholder orientation and firm performance, we found a direct relationship between employee orientation and firm performance, employee orientation and supply chain orientation, shareholder orientation and supply chain orientation as well as between supply chain orientation and firm performance. Hence our findings clarify how employee orientation, shareholder orientation and supply chain orientation influence firm performance. Accordingly, simultaneous attention to the interests of both internal and external stakeholder is required for increased firm performance.

Second our findings revealed that there are two direct paths to firm performance from shareholder orientation and supply chain orientation. The results also show that the link between shareholder orientation and firm performance is not only direct but can be indirect through supply chain orientation. More precisely, investing resources in creating shareholder wealth and satisfying stakeholder interest for firm performance can be done through two routes: either directly from shareholder orientation or through external stakeholders within the supply chain. This finding edifies the question of “how does internal shareholder orientation influence firm performance?” Hence, we show that high levels of firm performance are achievable through two paths allowing us to expand the current theoretical justification for the effect of shareholder orientation on performance in the context of OMCs.

Third, the findings of the mediation analyses deepen our appreciation of the nuanced role of supply chain orientation in influencing the link between employee orientation and firm performance, which, to date, is underexplored. By integrating into the path between employee orientation and firm performance, we uncovered the role of supply chain orientation as the missing process through which employee orientation leads to performance. Accordingly, we offer an extra process step in the shareholder orientation-performance nexus. By incorporating supply chain orientation in the shareholder orientation-performance path, we discover the role of supply chain orientation as the missing mechanism that clarifies the nexus between shareholder orientation and firm performance.

Fourth, the PLS-SEM findings highlight employee orientation as the most important predictor of supply chain orientation. However, the NCA findings suggest that shareholder orientation has the largest effect size on supply chain orientation, underscoring the importance of shareholders to successful supply chain strategy implementation. Also, the findings from both PLS-SEM and NCA underscore the importance of supply chain orientations strategy over employee orientation and shareholder orientation strategies. Thereby providing a more nuanced understanding of how a firm's multifaceted supply chain strategy that emphasizes the needs of its stakeholders (i.e., employees, shareholders, and supply chain partners) defines its success.

### 6.2 Managerial implications

Our research has significant implications for managers. Firstly, our findings are relevant to managers facing the quandary between choosing a strategy of promoting either an employee orientation, shareholder orientation or supply chain orientation for improved firm performance. Our findings show that whereas both shareholder orientation and supply chain orientation would lead to higher firm performance, employee orientation would not. Therefore, if the choice of a single orientation is available, firms can achieve higher levels of performance with shareholder orientation or supply chain orientation as they directly lead to greater levels of performance and do not need to do that through any underlying processes.

Secondly, we underscore the need for managers of OMCs to reflect on the complementarity of employee orientation and supply chain orientation when strategizing for higher levels of firm performance. Although a strategy of supply chain orientation leads to increased performance, a strategy of employee orientation alone does improve firm performance. Hence though shareholder orientation is important, managers must deploy it in combination with supply chain orientation to increase firm performance. However, our findings also revealed that employee orientation does not lead to firm performance. Hence, it must be deployed in combination with either supply chain orientation strategy or shareholder orientation, or both, for higher levels of firm performance. Accordingly, when planning strategy choice for firm performance, managers of OMCs are mindful of the interplay of internal stakeholder orientation (*i.e.,* employee orientation and shareholder orientation) and external stakeholder orientation (*i.e.,* supply chain orientation).

Third, the results of the mediation analysis suggest that higher levels of supply chain orientation enhance the effects of both employee orientation and shareholder orientation on firm performance. Hence, businesses should initiate programmes and activities that create and maintain good relationships with supply chain partners. Finally, the results further highlight the value of employee orientation and shareholder orientation strategies for achieving supply chain orientation besides the importance of employee orientation, shareholder orientation, and supply chain orientation. These findings have sweeping implications for the configuration and deployment of supply chain strategy. Hence, we advise managers to ensure that the minimum levels of these orientations that guarantee firm performance are always provided. However, since there are no additional benefits for raising the levels of these orientations beyond what is required, managers should invest to maintain only the levels of employee orientation, shareholder orientation and supply chain orientation that produce the required levels of firm performance.

### 6.3 Limitations and future research directions

Our findings should be applied cautiously, bearing in mind the following limitations. Firstly, the sample for this study was exclusive to a specific sector (i.e., OMCs) and country (Ghana). Therefore, any generalisation beyond this country's context should be approached with care. Accordingly, researchers should investigate the transferability of this finding beyond OMCs (in the manufacturing sector) or Ghana. Secondly, data were collected from only one group of firms (OMCs) with the petroleum downstream in Ghana. Therefore, future research may be carried on a more comprehensive sample of all downstream players to understand the degree to which the findings may vary based on group or locational factors. Thirdly, our study is based on associations rather than causality mainly because the cross-sectional design employed does not provide irrefutable proof of causality. The evidence should therefore be considered in line with hypothetical contentions and theorised relationships [[86], [87]], which could be improved upon through a longitudinal study.

## 7 Conclusions

The study investigated (a) the effect of employee orientation and shareholder orientation on supply chain orientation and (b) the effects of employee orientation, shareholder orientation, and supply chain orientation on firm performance. The mediating role of supply chain orientation in the relationship between (a) employee orientation and firm performance as well as (b) shareholder orientation, and firm performance were assessed. A multi-analytic approach involving the combination of both sufficiency (PLS-SEM) and necessity logic (NCA) were employed. The findings suggest that all predictors of supply chain collaboration (*i.e*., employee orientation and shareholder orientation) were significant. However, though the relationship between shareholder orientation and firm performance was not significant, both employee orientation and supply chain orientation significantly predict firm performance. Regarding the mediating role of supply chain orientation, the results reveal that supply chain orientation partially mediates the relationship between employee orientation and firm performance, it fully mediates the effect of shareholder orientation on firm performance. On the other hand, the findings from the necessary condition analysis suggest that all three orientations are necessary for firm performance. Putting everything together, the findings of this suggest that even though all three predictors of firm performance (*i.e.,* employee orientation, shareholder orientation and supply chain orientation) are necessary, only employee orientation and supply chain orientation are sufficient conditions for firm performance. The findings underscore the value of firm orientation to firm performance and demonstrate that whereas employee orientation and shareholder orientation are bottlenecks for firm performance, the three orientations also act as bottlenecks for firm performance.

## Author contribution statement

Innocent Senyo Kwasi Acquah: Conceived and designed the experiments; Performed the experiments; Analyzed and interpreted the data; Contributed reagents, materials, analysis tools or data; Wrote the paper.

Kassimu Issau; Rebecca Dei Mensah; Francis Vanderpuye: Performed the experiments; Contributed reagents, materials, analysis tools or data.

## Data availability statement

The authors do not have permission to share data.

## Declaration of competing interest

The authors declare that they have no known competing financial interests or personal relationships that could have appeared to influence the work reported in this paper.

## Figures and Tables

**Table:** Appendix A.: demographic characteristics of respondents

	Frequency
Age Group	
**20**–**29 years**	38
**30**–**39 years**	107
**40**–**49 years**	86
**50**–**59 years**	34
Total	265
Educational Qualification	
**Diploma/HND**	79
**Degree**	101
**Masters**	64
**PhD**	5
**Others**	16
Total	265
Gender	
**Male**	169
**Female**	96
Total	265
Working Experience	
**3**–**6 years**	83
**7**–**10 years**	67
**10**–**13 years**	74
**More than 13 years**	41
Total	265

**Table:** Appendix B.: Descriptive statistics for measurement items

Name	Mean	Standard deviation	Excess kurtosis	Skewness
*emo1*	3.923	0.693	5.951	−1.703
*emo2*	4.022	0.613	4.195	−1.030
*emo3*	3.834	0.811	1.318	−0.940
*emo4*	4.006	0.617	1.544	−0.572
*sho1*	3.917	0.680	2.191	−0.851
*sho2*	4.000	0.697	0.794	−0.592
*sho3*	4.055	0.679	0.245	−0.389
*sho4*	3.884	0.707	2.396	−0.869
*sho5*	3.878	0.601	1.290	−0.562
*sco1*	3.768	0.766	2.725	−1.211
*sco2*	3.818	0.669	1.190	−0.439
*sco3*	3.856	0.706	0.578	−0.547
*sco4*	3.796	0.741	2.054	−1.046
*sco5*	3.762	0.824	2.809	−1.387
*sco6*	3.878	0.748	1.981	−0.914
*fmp1*	4.083	0.696	4.622	−1.401
*fmp2*	4.044	0.655	4.855	−1.236
*fmp3*	3.983	0.660	1.384	−0.678
*fmp4*	3.923	0.685	1.231	−0.732

**Table:** Appendix C.: Total variance explained

Factor	Initial Eigenvalues			Extraction Sums of Squared Loadings		
	Total	% of Variance	Cumulative %	Total	% of Variance	Cumulative %
1	7.162	37.697	37.697	6.552	34.486	34.486
2	1.940	10.208	47.905			
3	1.626	8.558	56.463			
4	1.372	7.220	63.683			
5	1.120	5.895	69.577			
6	.760	4.000	73.577			
7	.741	3.903	77.480			
8	.652	3.429	80.909			
9	.599	3.152	84.061			
10	.528	2.780	86.841			
11	.435	2.288	89.129			
12	.379	1.995	91.124			
13	.328	1.726	92.850			
14	.298	1.569	94.419			
15	.265	1.393	95.812			
16	.239	1.259	97.071			
17	.217	1.140	98.210			
18	.182	.960	99.170			
19	.158	.830	100.000			

**Table:** Appendix D.: Endogeniety test using Gaussian copula approach

Test		Construct	Coefficient	P value
Gaussian copula approach	Endogenous variables			
Model 1	Employee Orientation	EMO ➔ FMP	0.713	0.020
		EMO ➔ SCO	0.365	0.000
		SCO ➔ FMP	0.301	0.005
		SHO ➔ FMP	0.269	0.004
		SHO ➔ SCO	0.331	0.000
		^GC^ (EMO) ➔ FMP	−0.509	0.073
Model 2	Supply Chain Orientation	EMO ➔ FMP	0.198	0.011
		EMO ➔ SCO	0.365	0.000
		SCO ➔ FMP	0.375	0.281
		SHO ➔ FMP	0.279	0.002
		SHO ➔ SCO	0.331	0.000
		^GC^ (SCO) ➔ FMP	−0.08	0.786
Model 3	Shareholder Orientation	EMO ➔ FMP	0.187	0.017
		EMO ➔ SCO	0.365	0.000
		SCO ➔ FMP	0.297	0.004
		SHO ➔ FMP	0.035	0.911
		SHO ➔ SCO	0.331	0.000
		^GC^ (SHO) ➔ FMP	0.256	0.364
Model 4	Employee Orientation,	EMO ➔ FMP	0.713	0.025
	Supply Chain Orientation	EMO ➔ SCO	0.365	0.000
		SCO ➔ FMP	0.302	0.392
		SHO ➔ FMP	0.269	0.004
		SHO ➔ SCO	0.331	0.000
		^GC^ (EMO) ➔ FMP	−0.509	0.083
		^GC^ (SCO) ➔ FMP	−0.001	0.997
Model 5	Employee Orientation,	EMO ➔ FMP	0.716	0.023
	Shareholder Orientation	EMO ➔ SCO	0.365	0.000
		SCO ➔ FMP	0.301	0.004
		SHO ➔ FMP	0.001	0.999
		SHO ➔ SCO	0.331	0.000
		^GC^ (EMO) ➔ FMP	−0.524	0.072
		^GC^ (SHO) ➔ FMP	0.281	0.352
Model 6	Supply Chain Orientation,	EMO ➔ FMP	0.186	0.018
	Shareholder Orientation	EMO ➔ SCO	0.365	0.000
		SCO ➔ FMP	0.427	0.224
		SHO ➔ FMP	0.020	0.951
		SHO ➔ SCO	0.331	0.000
		^GC^ (SCO) ➔ FMP	−0.133	0.657
		^GC^ (SHO) ➔ FMP	0.273	0.338
Model 7	Employee Orientation,	EMO ➔ FMP	0.710	0.030
	Shareholder Orientation,	EMO ➔ SCO	0.365	0.000
	Supply Chain Orientation	SCO ➔ FMP	0.355	0.315
		SHO ➔ FMP	−0.006	0.987
		SHO ➔ SCO	0.331	0.000
		^GC^ (EMO) ➔ FMP	−0.519	0.084
		^GC^ (SCO) ➔ FMP	−0.056	0.854
		^GC^ (SHO) ➔ FMP	0.288	0.346

Notes: EMO = Employee Orientation, SCO = Supply Chain Orientation, SHO = Shareholder Orientation, FMP = Firm performance, GC = Gaussian Copula.

**Table:** Appendix.: E1 Segement sizes

Number of Segments	Segment 1	Segment 2	Segment 3	Segment 4
1	1.000 (265)			
2	0.560 (149)	0.440 (116)		
3	0.437 (116)	0.422 (112)	0.141 (37)	
4	0.437 (116)	0.341 (90)	0.120 (32	0.102 (27)

**Table:** Appendix.: E2 FIMIX measures for segements

	Segment 1	Segment 2	Segment 3	Segment 4
AIC (Akaike's information criterion)	873.224	545.799	536.052	**533.956**
AIC3 (modified AIC with Factor 3)	880.224	560.799	**559.052**	564.956
AIC4 (modified AIC with Factor 4)	887.224	**575.799**	582.052	595.956
BIC (Bayesian information criterion)	895.613	**593.776**	609.618	633.110
CAIC (consistent AIC)	902.613	**608.776**	632.618	664.110
HQ (Hannan-Quinn criterion)	882.301	**565.250**	565.877	574.155
MDL5 (minimum description length with factor 5)	1041.171	**905.686**	1087.879	1277.723
LnL (LogLikelihood)	−429.612	−257.899	−245.026	−235.978
EN (normed entropy statistic)	0	**0.890**	0.767	0.7430
NFI (non-fuzzy index)	0	0.923	0.773	0.7300
NEC (normalized entropy criterion)	0	19.996	42.187	46.544

**Table:** Appendix F.: Test of quadratic effects

Relationship	Coefficient	STDEV	T statistics	P values	f^2^
EMO*EMO ➔ FMP	−0.065	0.044	1.478	0.139	0.020
EMO*EMO ➔ SCO	0.004	0.038	0.096	0.923	0.000
SHO*SHO ➔ FMP	0.001	0.061	0.019	0.985	0.000
SHO*SHO ➔ SCO	−0.046	0.041	1.122	0.263	0.050
SCO*SCO ➔ FMP	0.001	0.086	0.013	0.99	0.000
