# Supplementary material for: When stakeholder orientations matter: Modelling employee orientation, shareholder orientation and supply chain orientation as necessary and sufficient conditions for firm performance
Source: Heliyon. 2023 Sep 21;9(10):e20359. doi: 10.1016/j.heliyon.2023.e20359 (PMC10522997; doi:10.1016/j.heliyon.2023.e20359)
Supplement: Multimedia component 1 [file mmc1.docx]

**SECTION A: STAKEHOLDER ORIENTATION**

| **EMPLOYEE ORIENTATION** | | | | | | | |
| --- | --- | --- | --- | --- | --- | --- | --- |
|  | **1** | **2** | **3** | **4** | **5** | **6** | **7** |
| 1. we have regular staff meetings with employees. |  |  |  |  |  |  |  |
| 1. we have regular staff appraisals in which we discuss employees’ needs. |  |  |  |  |  |  |  |
| 1. we survey staff at least once a year to assess their work attitudes. |  |  |  |  |  |  |  |
| 1. we try to find out the true feelings of staff about their jobs. |  |  |  |  |  |  |  |
| **SHAREHOLDER ORIENTATION** | **1** | **2** | **3** | **4** | **5** | **6** | **7** |
| 1. we regularly carry-out public relations aimed at stakeholders |  |  |  |  |  |  |  |
| 1. our objectives are driven by creating shareholder wealth |  |  |  |  |  |  |  |
| 1. designated managers are responsible for satisfying shareholders’ interests. |  |  |  |  |  |  |  |
| 1. we regularly compare our share value with that of our competitors. |  |  |  |  |  |  |  |
| 1. We have regular meetings with shareholders. |  |  |  |  |  |  |  |

**SECTION B: SUPPLY CHAIN ORIENTATION**

| **CUSTOMERS ORIENTATION** | | **1** | **2** | | | **3** | **4** | **5** | | **6** | **7** | |
| --- | --- | --- | --- | --- | --- | --- | --- | --- | --- | --- | --- | --- |
| 1. we constantly monitor our commitment to serving customer needs as part of our value chain activities | |  |  | | |  |  |  | |  |  | |
| 1. we communicate information about customer experiences across all units. | |  |  | | |  |  |  | |  |  | |
| 1. we develop value chain strategies based on our understanding of customers’ needs. | |  |  | | |  |  |  | |  |  | |
| 1. we measure customer satisfaction systematically and frequently as a part of our value chain activities. | |  |  | | |  |  |  | |  |  | |
| 1. we disseminate data on customers satisfaction at all levels on a regular basis as a part of our value chain activities. | |  |  | | |  |  |  | |  |  | |
| 1. we help our customers be prepared for developments in their markets. | |  |  | | |  |  |  | |  |  | |
| 1. we try to discover additional needs of our customers of which they may be unaware. | |  |  | | |  |  |  | |  |  | |
| 1. we seek opportunities in areas where customers have difficulty expressing their needs. | |  |  | | |  |  |  | |  |  | |
| 1. we try to recognize customer needs, as a part of our value chain activities, before the majority of the market recognizes them | |  |  | | |  |  |  | |  |  | |
| 1. we extrapolate key trends to understand what customers will need in the future. | |  |  | | |  |  |  | |  |  | |
| **COMPETITOR ORIENTATION** | **1** | | | **2** | **3** | | **4** | **5** | **6** | | **7** |  |
| 1. we constantly monitor our commitment to understanding competitors as a part of our value chain activities. |  | | |  |  | |  |  |  | |  |  |
| 1. we communicate information about competitor across all units. |  | | |  |  | |  |  |  | |  |  |
| 1. we develop value chain strategies based on our understanding of competitors. |  | | |  |  | |  |  |  | |  |  |
| 1. we assess competitors systematically and frequently as a part of our value chain activities. |  | | |  |  | |  |  |  | |  |  |
| 1. we disseminate data on our competitor at all levels on a regular basis as a part of our supply chain activities. |  | | |  |  | |  |  |  | |  |  |
| 1. we understand our competitors, as a part of or value chain activities, to be prepared for developments in our markets. |  | | |  |  | |  |  |  | |  |  |
| 1. we try to discover additional actions of our competitors of which we may be unaware. |  | | |  |  | |  |  |  | |  |  |
| 1. We seek opportunities in areas as where our competitors have difficulty delivering to customers. |  | | |  |  | |  |  |  | |  |  |
| 1. we try to recognize competitor actions before the majority of the market recognizes them. |  | | |  |  | |  |  |  | |  |  |
| 1. we extrapolate key trends, to understand what competitors may do in the future. |  | | |  |  | |  |  |  | |  |  |

| **SUPPLIER ORIENTATION** | **1** | **2** | **3** | **4** | **5** | **6** | **7** |
| --- | --- | --- | --- | --- | --- | --- | --- |
| 1. we constantly monitor our commitment to understand suppliers |  |  |  |  |  |  |  |
| 1. we communicate information about suppliers across all units |  |  |  |  |  |  |  |
| 1. we develop value chain strategies based on our understanding of suppliers. |  |  |  |  |  |  |  |
| 1. we assess suppliers systematically and frequently as a part of our value chain activities. |  |  |  |  |  |  |  |
| 1. we disseminate data suppliers at all levels on a regular basis |  |  |  |  |  |  |  |
| 1. we understand our suppliers, as a part of our value chain activities |  |  |  |  |  |  |  |
| 1. we try to discover additional actions of our suppliers of which we may be unaware. |  |  |  |  |  |  |  |
| 1. we seek opportunities in areas where our suppliers have difficulty delivering to us. |  |  |  |  |  |  |  |
| 1. we try to recognize supplier actions before majority of the market recognizes them. |  |  |  |  |  |  |  |
| 1. we extrapolate key trends to understand what suppliers may do in the future. |  |  |  |  |  |  |  |

| **LOGISTICS ORIENTATION** | **1** | **2** | **3** | **4** | **5** | **6** | **7** |
| --- | --- | --- | --- | --- | --- | --- | --- |
| 1. we constantly monitor our commitment to understanding our logistics activities as a part of our value chain activities. |  |  |  |  |  |  |  |
| 1. we communicate information about our logistics activities across all units as a part of our value chain activities. |  |  |  |  |  |  |  |
| 1. we develop value chain strategies based on our understanding of our logistics activities. |  |  |  |  |  |  |  |
| 1. we assess our logistics activities systematically and frequently |  |  |  |  |  |  |  |
| 1. we disseminate data on our logistics activities at all levels on a regular basis |  |  |  |  |  |  |  |
| 1. we understand our logistics activities to be prepared for developments in our markets. |  |  |  |  |  |  |  |
| 1. we try to discover additional logistics possibilities of which we may be unaware. |  |  |  |  |  |  |  |
| 1. we seek opportunities in areas where our current logistics function has difficulty delivering to customers. |  |  |  |  |  |  |  |
| 1. we try to recognize logistics possibilities before the majority of the market recognizes them. |  |  |  |  |  |  |  |
| 1. we extrapolate key trends to understand what logistics activities we may need in the future. |  |  |  |  |  |  |  |

| **OPERATIONS ORIENTATION** | **1** | **2** | **3** | **4** | **5** | **6** | **7** |
| --- | --- | --- | --- | --- | --- | --- | --- |
| 1. we constantly monitor our commitment to understanding our operations management activities |  |  |  |  |  |  |  |
| 1. we communicate information about our operations management activities across all units |  |  |  |  |  |  |  |
| 1. we develop value chain strategies based on our understanding of our operations management activities. |  |  |  |  |  |  |  |
| 1. we assess our operations management activities systematically and frequently |  |  |  |  |  |  |  |
| 1. we disseminate data on our operations activities at all levels on a regular basis |  |  |  |  |  |  |  |
| 1. we understand our operations management activities to be prepared for developments in our markets. |  |  |  |  |  |  |  |
| 1. we try to discover additional operations management possibilities which we may be unaware. |  |  |  |  |  |  |  |
| 1. we seek opportunities in areas where our current operations management function has difficulty delivering for us. |  |  |  |  |  |  |  |
| 1. we try to recognize operations management possibilities before the majority of the market recognizes them. |  |  |  |  |  |  |  |
| 1. we extrapolate key trends to understand what operations management activities we may need in the future |  |  |  |  |  |  |  |

| **VALUE-CHAIN COORDINATION** | **1** | **2** | **3** | **4** | **5** | **6** | **7** |
| --- | --- | --- | --- | --- | --- | --- | --- |
| 1. we constantly monitor our coordination of value chain functions. |  |  |  |  |  |  |  |
| 1. we coordinate information about our value chain across all units. |  |  |  |  |  |  |  |
| 1. we coordinate strategies based on understanding of our value activities. |  |  |  |  |  |  |  |
| 1. we coordinate our value chain activities systematically and frequently. |  |  |  |  |  |  |  |
| 1. we coordinate data on our value chain activities, at all levels on a regular basis. |  |  |  |  |  |  |  |
| 1. we coordinate our value chain activities to be prepared for develops in our markets. |  |  |  |  |  |  |  |
| 1. we coordinate our value chain activities to try to discover additional possibilities of which we may be unaware. |  |  |  |  |  |  |  |
| 1. we coordinate opportunities in areas where our value chain function has difficulty delivering for us. |  |  |  |  |  |  |  |
| 1. we try to coordinate value chain possibilities before majority of the market recognizes them. |  |  |  |  |  |  |  |
| 1. we extrapolate key trends to coordinate what value chain activities we may need in the future. |  |  |  |  |  |  |  |

**SECTION C: FIRM PERFORMANCE**

| **CUSTOMER PERFORMANCE** | **1** | | **2** | | **3** | | **4** | | **5** | | **6** | | **7** | | |
| --- | --- | --- | --- | --- | --- | --- | --- | --- | --- | --- | --- | --- | --- | --- | --- |
| 1. we achieved a high degree of customer satisfaction in the last year. |  | |  | |  | |  | |  | |  | |  | | |
| 1. we kept a large number of existing customer market share in the last year. |  | |  | |  | |  | |  | |  | |  | | |
| 1. we attracted a significant number of new customers in the last year. |  | |  | |  | |  | |  | |  | |  | | |
| 1. we secured a large portion of our desired market share in the last year. |  | |  | |  | |  | |  | |  | |  | | |
| **FINANCIAL PERFORMANCE** | | **1** | | **2** | | **3** | | **4** | | **5** | | **6** | | **7** | |
| 1. we achieved revenues above our stated objective in the last year. | |  | |  | |  | |  | |  | |  | |  | |
| 1. we achieved sales above our stated objective in the last year. | |  | |  | |  | |  | |  | |  | |  | |
| 1. we achieved return on investments above our stated objective in the last year. | |  | |  | |  | |  | |  | |  | |  | |
| 1. we achieved return on our assets above our stated objective in the last year. | |  | |  | |  | |  | |  | |  | |  | |
| **INTERNAL PROCESS PERFORMANCE** | | **1** | | **2** | | **3** | | **4** | | **5** | | **6** | | **7** | |
| 1. the speediness of our supply chain processes improved in the last year. | |  | |  | |  | |  | |  | |  | |  | |
| 1. the quality of our supply chain processes improved in the last year. | |  | |  | |  | |  | |  | |  | |  | |
| 1. the cost of our supply chain processes improved in the last year. | |  | |  | |  | |  | |  | |  | |  | |
| 1. the flexibility of our supply chain processes improved in the last year. | |  | |  | |  | |  | |  | |  | |  | |
| **INNOVATION AND LEARNING PERFORMANCE** | **1** | | **2** | | **3** | | **4** | | **5** | | **6** | | **7** | |  |
| 1. we significantly enhanced our marketing skills compared with last year. |  | |  | |  | |  | |  | |  | |  | |  |
| 1. we significantly enhanced our logistics skills compared with last year. |  | |  | |  | |  | |  | |  | |  | |  |
| 1. we significantly enhanced our supply management skills compared with last year. |  | |  | |  | |  | |  | |  | |  | |  |
| 1. we significantly enhanced our operations management skills compared with last year. |  | |  | |  | |  | |  | |  | |  | |  |

**SECTION D: BUSINESS INFORMATION**

**Please tick the appropriate box which is applicable in each question**

1. Which of the following best describes the legal form of your business?

Sole trader Partnership Company Joint Venture

1. How long have you been working for this company?

3-6 years 7-10 years 10-13 years more than 13 years

1. What is your position in the firm?

Operations Management Supply Chain Manager Logistics Manager

**SECTION E: DEMOGRAPHIC INFORMATION**

1. In which one of the following age groups are you?

Up to 19 years 20-29 years 30-39 years 40-49 years

50-59 years Above 60 years

1. What is your highest educational qualification?

WASSCE Diploma/HND Degree Masters

PhD Others

1. Gender Male Female
